# Mortality is associated with inflammation, anemia, specific diseases and treatments, and molecular markers

**DOI:** 10.1371/journal.pone.0175909

**Published:** 2017-04-19

**Authors:** Mark Moeller, Christiane Pink, Nicole Endlich, Karlhans Endlich, Hans-Jörgen Grabe, Henry Völzke, Marcus Dörr, Matthias Nauck, Markus M. Lerch, Rüdiger Köhling, Birte Holtfreter, Thomas Kocher, Georg Fuellen

**Affiliations:** 1 Institute for Biostatistics and Informatics in Medicine and Ageing Research, Rostock University Medical Center, Rostock, Germany; 2 Department of Restorative Dentistry, Periodontology, Endodontology, and Preventive and Pediatric Dentistry, University Medicine Greifswald, Greifswald, Germany; 3 Department of Anatomy and Cell Biology, University Medicine Greifswald, Greifswald, Germany; 4 Clinic of Psychiatry, Ernst Moritz Arndt University Greifswald, Greifswald, Germany; 5 Department of Study of Health in Pomerania/Clinical-Epidemiological Research, Institute for Community Medicine, University Medicine Greifswald, Greifswald, Germany; 6 German Centre for Cardiovascular Research (DZHK), partner site Greifswald, Greifswald, Germany; 7 German Centre for Diabetes Research (DZD), partner site Greifswald, Greifswald, Germany; 8 Department of Internal Medicine B, University Medicine Greifswald, Greifswald, Germany; 9 German Centre for Cardiovascular Research (DZHK), partner site Greifswald, Greifswald, Germany; 10 Institute of Clinical Chemistry and Laboratory Medicine, Ernst Moritz Arndt University Greifswald, Greifswald, Germany; 11 Department of Internal Medicine A, University Medicine Greifswald, Greifswald, Germany; 12 Institute of Physiology, Rostock University Medical Center, Rostock, Germany; Kaohsiung Medical University Hospital, TAIWAN

## Abstract

Lifespan is a complex trait, and longitudinal data for humans are naturally scarce. We report the results of Cox regression and Pearson correlation analyses using data of the Study of Health in Pomerania (SHIP), with mortality data of 1518 participants (113 of which died), over a time span of more than 10 years. We found that in the Cox regression model based on the Bayesian information criterion, apart from chronological age of the participant, six baseline variables were considerably associated with higher mortality rates: smoking, mean attachment loss (i.e. loss of tooth supporting tissue), fibrinogen concentration, albumin/creatinine ratio, treated gastritis, and medication during the last 7 days. Except for smoking, the causative contribution of these variables to mortality was deemed inconclusive. In turn, four variables were found to be associated with decreased mortality rates: treatment of benign prostatic hypertrophy, treatment of dyslipidemia, IGF-1 and being female. Here, being female was an undisputed causative variable, the causal role of IFG-1 was deemed inconclusive, and the treatment effects were deemed protective to the degree that treated subjects feature better survival than respective controls. Using Cox modeling based on the Akaike information criterion, diabetes, mean corpuscular hemoglobin concentration, red blood cell count and serum calcium were also associated with mortality. The latter two, together with albumin and fibrinogen, aligned with an”integrated albunemia” model of aging proposed recently.

## Introduction

Despite its importance in a world of rapid demographic change towards an increasing proportion of elderly citizens, we do not understand in detail what aging is, nor do we understand what is cause and what is consequence of aging; i.e. which marker changes are causal to aging and which ones are just the consequences of the aging process. Nevertheless, investigations into cause and consequence in the human system require a set of hypotheses to begin with. Large-scale population studies are one source for such hypotheses, and the Study of Health in Pomerania (SHIP) [1, 2] is emerging as a rich source of marker observations, including mortality data. In fact, the first cohort is now undergoing its third follow up, and as of 19.08.2011, for 567 out of 4308 participants that were recruited between 1997 and 2001, it is known that they died, and when. This data enables a detailed study of the relationships between SHIP variables and mortality / survival.

Naturally, the results of any modeling of mortality strongly depend on the set of input variables, on the methodology (such as Cox proportional hazards modeling), and on the population under study. Input variables may be omics data, established markers related to life-style, clinical chemistry laboratory data, disease symptoms or disease diagnosis and treatment, and/or socio-demographic data. The populations under investigation may be representative of large segments of the entire population of a geographic region, or there may be a focus on, e.g., the oldest old. Genetic data afford genome-wide association studies of any traits that are also measured in the population sample, while gene (or protein, or metabolite) expression data may also allow deep molecular mechanistic insights into mortality determinants such as hypotheses about pathway activations or inhibitions related to mortality. Laboratory data allow such mechanistic insights on a more aggregative level; e.g. anemia, inflammation, immunity or growth can be estimated by specific markers such as blood cell counts. On the most aggregative level, very general traits and socio-demographic attributes such as chronological age, gender, education, income or life style risk factors (smoking, alcohol consumption, physical activity) were shown in the past to have a strong influence on mortality [3].

Of all the laboratory, diagnosis / treatment and socio-demographic data available in the SHIP study we considered 77 variables with *complete* data records for 1518 participants, of which 113 had been recorded dead during follow-up. Thus, the studies closest to ours are mortality studies of older people with similar input data. Specifically, Cohen et al. [4] integrated data describing 43 common clinical biomarkers from three longitudinal cohort studies (Women’s Health and Aging I & II, InCHIANTI, and the Baltimore Longitudinal Study on Aging). Using principal component analysis (PCA) of the variables they revealed a strong role of markers of anemia and inflammation, which together with calcium und albumin dominated the first PCA axis, while the second axis was related to metabolic syndrome. The relationship between PCA axes and mortality was demonstrated using Cox models. Similarly, Walter et al. [5] analyzed mortality in the Rotterdam study. Their Cox modeling revealed that mortality could be explained, jointly and in individual associations, by chronological age and gender, but also by physiological markers such as body mass index and leucocyte count, by prevalent diseases such as cancer, and by general health parameters such as self-assessed health, and memory complaints. Notably, they found that 6 (out of 93) genetic markers were also able to explain mortality in part, but even jointly, these contributed little to mortality prediction. Self-assessment of health was also found important in a Cox analysis of SHIP data [6], where input was restricted to three subjective health assessment scores, ten molecular markers, and some socio-demographics using data from 4264 participants including 456 deaths. They found that a combination of self-assessment and biomarkers enabled best mortality predictions. The Newcastle 85+ study [7] analyzed data of 719 individuals aged 85 at time of recruitment, 50 of which had died after an 18-month follow up (Martin-Ruiz, Personal Communication, February 10, 2016), employing a panel of 74 markers. Twenty-four of these markers associated significantly with mortality in multivariate models, among them a variety of laboratory markers. The strongest associations with the best significance were based on total protein, plasma N-terminal pro-B-type natriuretic peptide (BNP), and vitamin B6 status. The Vitality 90+ study [8] investigated and integrated conventional predictors (including cell free DNA levels as molecular markers, and blood pressure measurements as physiological markers) and gene expression, for a sample of 151 people (49 of which had died) who were exactly 90 years old at time of recruitment. They found that in multivariate models, body-mass index, cell free DNA, and frailty were predictive of mortality, as were some gene-expression-based molecular pathways. When combined, body-mass index, frailty and 9 gene expression values were predictive, where the genes showed close proximity to the inflammation master regulator NFkB, in an Ingenuity pathway analysis. Survival to age 90 was studied in a Swedish sample of 380 75-year-olds, of which 234 had died before the age of 90 [9]. Significant predictors of survival were variables based on an exercise test, on lung and heart function tests, BNP, HDL-cholesterol, heart-related diagnoses (men only), and white blood cell counts (women only). Mortality was also studied in an Australian population of men aged 70 and older (the Concord study), 461 of which had died [10]. Here, Cox modeling revealed that chronological age, smoking status, some laboratory values (white blood cell count, anemia and albumin), various disease diagnoses as well as body mass index (BMI) and physical fitness were predictive.

Overall, there is a wide heterogeneity in findings, which can be explained, in part, by the specific population sample, the methodology, and the number and kind of markers that were measured in the first place. Moreover, the relevance of findings for designing interventions is the higher the closer these reflect causal and amenable contributions to mortality. Thus, it is important to scrutinize cohort studies for markers of mortality, to identify common patterns in the resulting findings, and to investigate causality.

In this report, we confirmed well-established markers, and we specifically confirmed, to a large part, a recently proposed physiologically defined aging process underlying mortality, i.e. “integrated albuminemia” [4]. We also aimed for a delineation of predictive markers by the knowledge we could find in the literature regarding their contribution to the aging-associated processes that are behind mortality. For many markers, bidirectional associations with aging might be assumed; our label for these markers is “inconclusive causation”, and the complexity of biological processes suggests that such inconclusiveness is the default attribute. Moreover, given a predictive marker and its relationship to aging and mortality, we never know whether there may be unknown influences that drive both the marker, and aging and mortality. Nevertheless, to understand the aging process and to propose and evaluate possible interventions, it is mandatory to tackle associations to the best of our abilities.

## Methods

The Study of Health in Pomerania (SHIP) [1, 2] is a population-based longitudinal health survey. It aims to describe the prevalence and distribution of a broad range of diseases, as well as environmental and behavioral risk factors in the region of Western Pomerania (North-Eastern Germany). The main aim was to investigate health in all its aspects and complexity. Based on a two-stage cluster design, three cities and 12 larger towns were selected and 17 smaller villages were randomly drawn. From these, a sample of 7006 Caucasian subjects (age 20–79 years) was drawn via population registries. Within this sample, there were 741 losses (126 subjects deceased and 615 migrated). Finally, 4308 (2116 males and 2192 females) of 6265 eligible subjects participated in the baseline examinations from 1997 to 2001 (SHIP-0). The data collection and instruments included four parts: an oral health examination, a medical examination, a health-related interview, and a heaIth- and risk- factor-related questionnaire. The oral health examination included the teeth, periodontium, oral mucosa, craniomandibular system, and prosthodontics. The medical examination included standardized blood pressure measurements, electrocardiography, echocardiography, carotid, thyroid and liver ultrasounds, neurological screening, blood and urine sampling. The computer-aided health-related interview included cardiovascular symptoms, utilization of medical services, health-related behaviors, and socioeconomic variables. The self-administered questionnaire comprised housing conditions, social network, work conditions, subjective well-being and individual consequences from the German reunification. Further details on the study and processing of data can be obtained elsewhere [1]. SHIP was approved by the Ethical Review Board of the University of Greifswald and was carried out according to WMA declaration of Helsinki. All participants gave written consent.

### Statistical analyses

We studied variables measured in the SHIP cohort that may qualify as predictive markers of aging. Our main aim was thus to identify those SHIP variables that best model mortality / survival of the study participants. In SHIP, not only quantitative data was gathered, but also qualitative information on diseases and their treatments and other variables, and we decided to use the Cox proportional hazards modeling approach [11]. A list of all variables that were considered for modeling can be found in Table A in S1 File.

Specifically, 77 variables were preselected based on the "Data Dictionary SHIP-0" (https://www.fvcm.med.uni-greifswald.de/dd_service/data_use_intro.php), which overall lists a few thousand variables, including a very detailed description of dental status. For selection, we considered the following expert selection criteria:

- *Be parsimonious*. Access to SHIP data must be justified; it is not appropriate to "ask for all the data" for purely explorative analyses because if the amount of data exceeds a certain size, re-identification issues may surface. Moreover, SHIP rules for data access were interpreted strictly in our case, because the data were shared between two research groups, one of which (Rostock) working with SHIP data for the first time.
- *Avoid overfitting and multicollinearity*. If model fitting processes start with a high ratio of subjects to variables, they may be prone to overfitting. In case of multiple closely related variables from the Data Dictionary, we selected the one that was better known, and/or that was used by us and other researchers working with SHIP data in the past. (For the correlation matrix see Figure A in S1 File.)
- *Exclude problematic variables*. As described in the Data Dictionary and other SHIP documentation, for some variables, artefacts such as systematic measurement errors are known.
- *Strive for relevance*. Finally, we included variables related to periodontitis based on our specific interest in these. Also, we restricted ourselves to variables for which we could expect a relationship with mortality.

**Fig 1:**
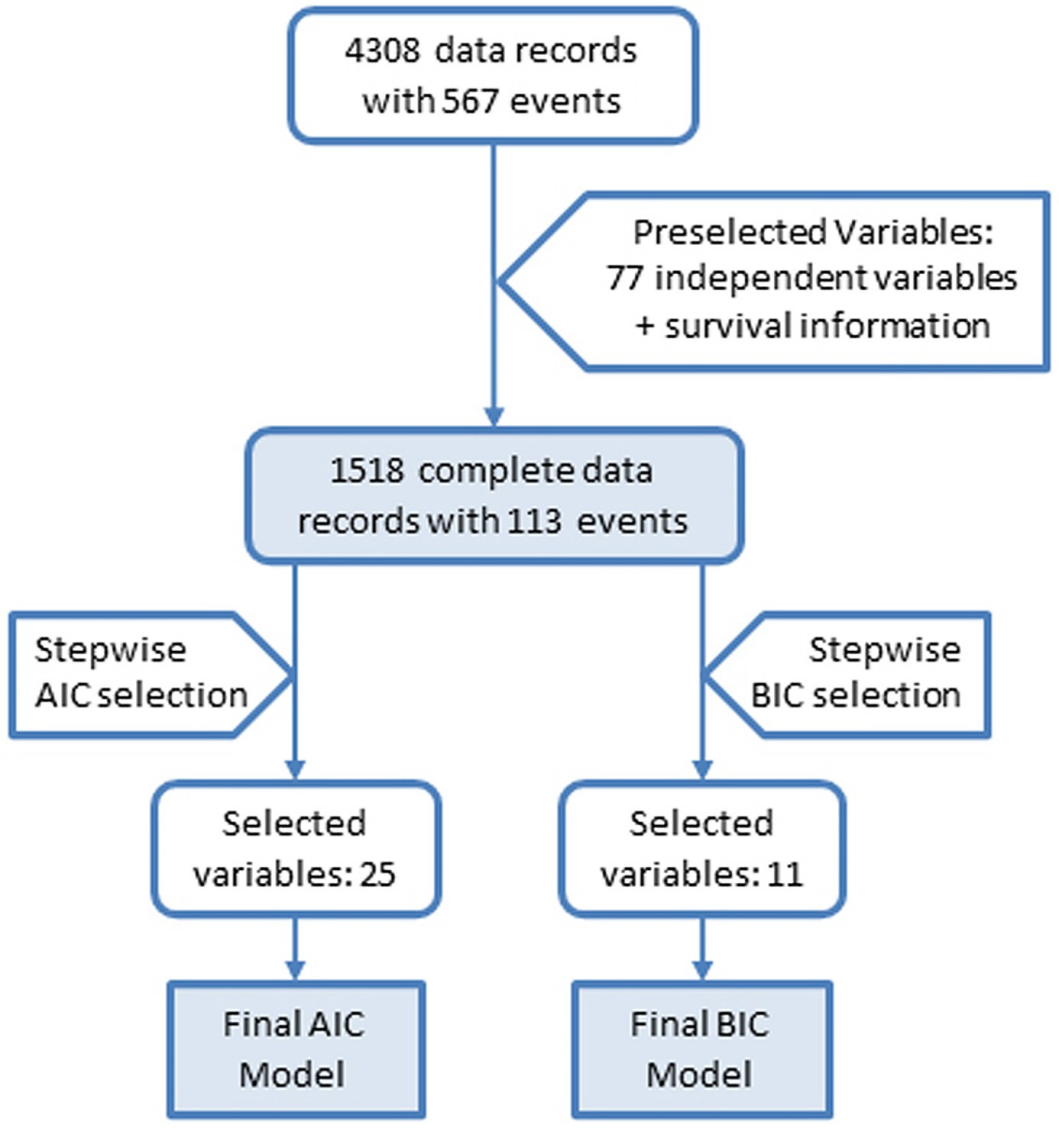
Flowchart for the building of the AIC and the BIC Model. We started with 4308 data records, 77 preselected variables and two variables for “total mortality” and “follow-up time”, which are the status indicator and the follow-up time for the right-censored mortality data. Thereafter we obtained 1518 complete data records with these variables. Based on AIC and BIC criteria, we obtained two different models with 25 and 11 variables, respectively, referred to as AIC and BIC models.

We used Cox regression, and specifically the Akaike information criterion (AIC) and the Bayesian information criterion (BIC) to guide model selection [12]. Cox regression does not allow any missing values, and we did no imputing. As described, seventy-seven variables were preselected; yielding 1518 complete data records with 113 death events (see Fig 1), and our preselection aimed at the most promising candidates to study the relationship of markers (molecular, lifestyle and disease-related) and mortality. The molecular markers are listed under “Laboratory variables” in Table A in S1 File. We used the R environment for statistical computing version 3.2.2 [13] as the framework for the analyses, and its *survival* package [14, 15] for the Cox analyses. Additionally, we performed correlation analyses for each variable against the age of the participant at baseline. This correlation gives an idea on the average trajectory of the single variables over the lifespan of the participants.

Except for age, continuous variables were standardized to z-scores as z-score = (original value − mean) / standard deviation. We then built one AIC model and one BIC model. Baseline information on the variables selected for these two models can be found in Table 1. More specifically, we used the stepAIC function of the MASS package [16]; the algorithm parameter *direction* was set to “both”, so that, coming from the complete model, it tests if skipping one variable at each iteration increases the maximum likelihood for this model and after each step it checks whether the addition of a formerly skipped variable improves the model with respect to the maximum likelihood of the model. Additionally, we set the penalty parameter *k* to *k = 2* to obtain an optimal AIC model, and to *k = log (n)* with *n* being the number of data records to obtain the best BIC model, respectively.

**Table 1:** Baseline characteristics of variables appearing in the final AIC and BIC models (N = 1518).

Variable	Mean ± SD or N (%)	Reference values, if applicable^a^
Age, years	46.3 ± 15.5	
Female sex	782 (51.5%)	
Smoking status		
Never smoker	540 (35.6%)	
Ex-smoker	496 (32.7%)	
Current smoker	482 (31.8%)	
Household equivalent income, €	981.4 ± 483.3	
SF-12 sum-score of physical health	49.1 ± 8.2	100: best health score
Mean attachment loss, mm	2.65 ± 1.95	
Depression, yes	189 (12.5%)	
Diabetes mellitus, yes	124 (8.2%)	
Treated dyslipidemia, yes	166 (10.9%)	
Treated gastritis, yes	82 (5.4%)	
Treated osteoporosis, yes	47 (3.1%)	
Number of drugs taken within the last 7 days	1.77 ± 2.13	
Antihypertensives, yes	333 (21.9%)	
Sex hormones and modulators of the genital system, yes	270 (17.8%)	
HMG CoA reductase inhibitors, yes	85 (5.6%)	
Drugs used in benign prostatic hypertrophy, yes	51 (3.4%)	
Serum calcium, mmol/l	2.4 ± 0.1	2.15–2.50 mmol/l
Ferritin, μg/l	96.6 ± 105.6	f: 10–150 μg/l; m: 30–300 μg/l
IGF-1, ng/ml	150.2 ± 59.4	strongly age dependent
Fibrinogen, g/l	2.9 ± 0.7	1.5–3.5 g/l
Albumin/creatinine ratio, mg/mmol	21.9 ± 68.0	< 3 mg/mmol
Red blood cell count, Tpt/l	4.4 ± 0.4	f: 3.7–5.0 Tpt/l; m: 4.2–5.5 Tpt/l
Hemoglobin, mmol/l	8.4 ± 0.8	7.5–11 mmol/l
Mean corpuscular hemoglobin concentration, mmol/l	21.2 ± 0.6	19.9–22.3 mmol/l

^a^source: Merck Manual (Professional Version). Continuous variables are described by mean ± standard deviation (SD); categorical variables are described by Number (%). IGF-1, Insulin like growth factor 1

## Results

We obtained one AIC model with 25 variables (Table 2) and one smaller BIC model with eleven variables (Table 3). The BIC model was smaller, as expected, due to the heavier penalization of model complexity in case of BIC. Both models were nested; all variables of the BIC-model were also present in the AIC model. Because a sufficient number of events per variable is needed to avoid a bias of the Cox regression coefficients [17–19], the BIC model with eleven variables and 113 events was, all things being equal, preferable to the AIC model. The proportional hazard assumption was fulfilled for all variables in both models. R squared was 0.22 for the AIC model and 0.20 for the BIC model.

**Table 2:** Results for the AIC Model based on 1518 subjects with 113 events.

Variable	HR (95% CI)	P-value
Age, years	1.09 (1.06; 1.12)	<0.01
Female sex	0.47 (0.28; 0.79)	<0.01
Smoking status (Ref. Never smoker)		
Ex-smoker	1.51 (0.87; 2.62)	0.14
Current smoker	3.55 (1.89; 6.65)	<0.01
Household equivalent income, € ^**a**^	0.77 (0.59; 1.01)	0.06
SF-12 sum-score of physical health ^**a**^	0.87 (0.73; 1.03)	0.11
Mean attachment loss, mm ^**a**^	1.47 (1.23; 1.75)	<0.01
Depression, yes	1.65 (0.86; 3.15)	0.13
Diabetes mellitus, yes	1.63 (1.02; 2.60)	0.04
Treated dyslipidemia, yes	0.29 (0.16; 0.55)	<0.01
Treated gastritis, yes	2.48 (1.31; 4.71)	0.01
Treated osteoporosis, yes	0.48 (0.18; 1.28)	0.14
Number of drugs taken within the last 7 days	1.17 (1.05; 1.29)	<0.01
Antihypertensives, yes	0.69 (0.42; 1.12)	0.13
Sex hormones and modulators of the genital system, yes	0.38 (0.09; 1.65)	0.19
HMG CoA reductase inhibitors, yes	1.70 (0.89; 3.26)	0.11
Drugs used in benign prostatic hypertrophy, yes	0.40 (0.21; 0.77)	0.01
Serum calcium, mmol/l ^**a**^	1.43 (1.15; 1.77)	<0.01
Ferritin, μg/l ^**a**^	0.85 (0.69; 1.04)	0.12
IGF-1, ng/ml ^**a**^	0.63 (0.46; 0.85)	<0.01
Fibrinogen, g/l ^**a**^	1.53 (1.30; 1.78)	<0.01
Albumin/creatinine ratio, mg/mmol ^**a**^	1.16 (1.05; 1.27)	<0.01
Red blood cell count, Tpt/l ^**a**^	0.54 (0.37; 0.78)	<0.01
Hemoglobin, mmol/l ^**a**^	1.44 (0.97; 2.15)	0.07
Mean corpuscular hemoglobin concentration, mmol/l ^**a**^	0.67 (0.54; 0.84)	<0.01

Z-standardized continuous variables are marked by (^a^).

HR, hazard ratio; CI, confidence interval. IGF-1, Insulin like growth factor 1

**Table 3:** Results for the BIC Model based on 1518 subjects with 113 events.

Variable	HR (95% CI)	P-value
Age, years	1.09 (1.06; 1.11)	<0.01
Female sex	0.51 (0.32; 0.80)	<0.01
Smoking status (Ref. Never smoker)		
Ex-smoker	1.44 (0.85; 2.43)	0.17
Current smoker	3.37 (2.13; 6.55)	<0.01
Mean attachment loss, mm ^**a**^	1.46 (1.23; 1.72)	<0.01
Treated dyslipidemia, yes	0.40 (0.22; 0.70)	<0.01
Treated gastritis, yes	2.48 (1.34; 4.61)	<0.01
Number of drugs taken within the last 7 days	1.20 (1.11; 1.30)	<0.01
Drugs used in benign prostatic hypertrophy, yes	0.38 (0.20; 0.73)	0.01
IGF-1, ng/ml ^**a**^	0.69 (0.52; 0.92)	0.01
Fibrinogen, g/l ^**a**^	1.46 (1.27; 1.68)	<0.01
Albumin/creatinine ratio, mg/mmol ^**a**^	1.16 (1.06; 1.26)	<0.01

Z-standardized continuous variables are marked by (^a^).

HR, hazard ratio; CI, confidence interval

Examining the AIC model (Table 2) we observed several variables with p-values larger than 0.05. These variables are important for the mathematical model, but since they are statistically insignificant they will be not considered as relevant and not be discussed further. Both the AIC (Table 2) and the BIC model (Table 3) include seven variables (with p<0.05) for which an increase in value resulted in an increased hazard ratio (age at baseline, smoking, mean attachment loss, treated gastritis, the number of drugs taken within the last 7 days, fibrinogen concentration, and albumin/creatinine ratio). Additionally, serum calcium concentration and diabetes mellitus (p<0.05) were included in the AIC model. On the other hand, there are four variables in both models with a decreasing effect on the hazard ratio if their value is increased (intake of drugs to treat benign prostatic hypertrophy, treated dyslipidemia, IGF-1 and female sex). The AIC model included two additional variables where an increase in value resulted in a significantly decreased hazard ratio: red blood cell count and mean corpuscular hemoglobin concentration (p<0.05).

Both models are juxtaposed in Table 4. To better interpret the single variables, we calculated variable correlations to age at baseline (that is, chronological age), estimated their significance, and sorted the variables by their correlation coefficient. Moreover, in the column “Kind of marker”, we classified the variables by their putative causal relationship to mortality, as described in detail in the Discussion section. A detailed comparison of hazard ratios for the two models is given in Table B in S1 File (where continuous variables were z-standardized) and Table C in S1 File (where continuous variables were not z-standardized).

**Table 4:** Comparison of the AIC and BIC model and correlation analysis (with P-value) of variables against age at baseline (orange: positive correlation, grey: negative correlation; white: correlation not statistically significant).

	Hazard Ratio		Kind of marker	All (N = 1518)		Females (N = 782)		Males (N = 736)	
Variable	AIC Model	BIC Model		Corr	P-value	Corr	P-value	Corr	P-value
Mean attachment loss, mm*	1.47	1.46	Inconclusive causation	0.65	<0.01	0.67	<0.01	0.64	<0.01
Number of drugs taken in last 7 days	1.17	1.20	Inconclusive causation	0.49	<0.01	0.42	<0.01	0.57	<0.01
Diabetes mellitus, yes	1.63	-	Inconclusive causation	0.28	<0.01	0.27	<0.01	0.28	<0.01
Drugs used in benign prostatic hypertrophy, yes	0.40	0.38	Treatment advantage	0.27	<0.01			0.36	<0.01
Fibrinogen, g/l ^**a**^	1.53	1.46	Inconclusive causation	0.27	<0.01	0.21	<0.01	0.33	<0.01
Treated dyslipidemia, yes	0.29	0.40	Treatment advantage	0.26	<0.01	0.29	<0.01	0.22	<0.01
Albumin/creatinine ratio, mg/mmol ^**a**^	1.16	1.16	Inconclusive causation	0.13	<0.01	0.10	<0.01	0.18	<0.01
Treated gastritis, yes	2.48	2.48	Inconclusive causation						
Mean corpuscular hemoglobin concentration, mmol/l ^**a**^	0.67	-	Inconclusive causation						
Red blood cell count, Tpt/l ^**a**^	0.54	-	Inconclusive causation	-0.06	0.02	0.09	0.01	-0.29	<0.01
Serum calcium, mmol/l ^**a**^	1.43	-	Inconclusive causation	-0.08	<0.01			-0.22	<0.01
Female sex	0.47	0.51	Undisputed risk marker	-0.09	<0.01				
Current smoker	3.55	3.73	Undisputed risk marker	-0.29	<0.01	-0.27	<0.01	-0.32	<0.01
IGF-1, ng/ml ^**a**^	0.63	0.69	Inconclusive causation	-0.49	<0.01	-0.49	<0.01	-0.50	<0.01

Z-standardized continuous variables are marked by (^a^).

Corr: correlation; for the “Kind of marker” classification see Discussion.

## Discussion

Overall, Cox modeling of SHIP mortality data highlighted many risk markers already known to be correlated with aging processes. Some of these risk markers are also part of established causal explanations of aging, although in most cases we assume that due to the complexity of human physiology and due to feedback loops in particular, quite often “the distinction between correlation and causation collapses” [20]; our term for these markers will be “inconclusive-causation markers”.

We thus distinguish four classes of markers: (1) undisputed risk markers; these are chronological age, smoking status and sex; (2) inconclusive–causation markers; these are attachment loss (reflecting periodontitis), diabetes mellitus, treated gastritis, and the number of drugs taken within the last 7 days; (3) advantage-of-treatment markers, where the medical care may provide an advantage, highlighting benign prostatic hypertrophy and dyslipidemia; and (4) blood/urine-based inconclusive-causation markers (fibrinogen, red blood cell count, albumin/creatinine ratio, serum calcium, IGF1, mean corpuscular hemoglobin concentration), associated with inflammation [21], “integrated albunemia” [4], and frailty [22]. In the following, model variables with p<0.05 in the respective prediction models are discussed. Comparing the AIC and the BIC model, hazard ratios as well as p-values are remarkably consistent, so that we discuss variables irrespective of the kind of model; the reader may note that some variables are only picked up by the AIC model, though.

### Undisputed risk markers

*Age at baseline* (chronological age) was clearly relevant and predictive in our models. This is in line with previous reports, proposing chronological age to be the major risk marker for several diseases with high mortality rates, including cancer, stroke and heart disease [23]. In line with our models, s*moking* was identified as relevant and predictive of mortality already in the last century, as it implies damage to the body in many aspects, specifically resulting in higher risk of COPD or cancer [24]. It further increases mortality hazard at all ages [25–27]. Finally, being of female *sex* decreased mortality risk significantly (HR = 0.47 for the AIC model and HR = 0.45 for the BIC model); the different biological and social aspects of sex bias in mortality were already described in great detail [28].

### Inconclusive-causation markers

*Clinical attachment loss* reflects lifetime accumulated periodontal disease experience. In previous studies using SHIP data, destructive periodontitis was shown to have bidirectional associations with low grade systemic inflammation [29, 30] and with diabetes [31–33]. *Attachment loss* is a prototypical inconclusive-causation marker that may reflect worsened physical conditions. On the one hand, inflammatory processes are attributed to the aging process (“inflammaging”, [21]), and general inflammatory processes most likely contribute causally to increasing inflammation of the periodontium in particular [29]. On the other hand, it is plausible that inflammation of the periodontium affects the entire body via low grade systemic inflammation [34], causing an acceleration of aging-related processes [35]. In support of this statement, chronic periodontitis was shown to impact the risk of stroke [36], myocardial infarction [37], rheumatoid arthritis [38], and cardiovascular and all-cause mortality [39, 40].

A diagnosis of *diabetes mellitus* is known to increase mortality risk significantly [41], and again, an inconclusive chicken-and-egg scenario of causation is most plausible, where the metabolic imbalance that is the hallmark of diabetes drives aging processes (cellular damage in particular), and core aging processes including (oxidative) damage (beyond hormetic amounts) worsen the diabetic state.

In our models, *treated gastritis* was predictive of mortality. For the treatment of gastritis either histamine 2 receptor antagonists (H2RA) or proton pump inhibitors (PPI) are widely used as either prescription or over-the-counter medications in Germany. The SHIP study area has the highest per capita consumption of PPI in Germany, but the original study questionnaire did not differentiate separately between these drug classes. *Treated gastritis* could therefore be a surrogate for the use of H2RA or PPI, both of which have been linked to increased mortality either individually or in combination with other drugs, specifically the antidiabetic agent metformin [42]. The underlying therapeutic principle as well as the risk-conferring mechanism for both classes of drugs is the neutralization of intragastric pH. PPI treatment suppresses the split of proteins and therefore has an impact on protein metabolism, supporting malnutrition and sarcopenia. Also, reducing gastric acidity greatly impairs the elimination of infectious agents from the stomach and gastrointestinal tract. Not unexpectedly a variety of population-based and case control studies have linked the use of PPI to increased rates of infectious diseases [43, 44] including respiratory, gastrointestinal and intraperitoneal infections depending on the prevalent comorbidities [45–47]. This may explain the association of an increased mortality with the prior treatment of gastritis, without having to point to any specific cause of death.

Finally, a very plausible inconclusive-causation marker is the *number of drugs taken in the last 7 days*. Polypharmacy is a natural consequence of aging and disease, as it reflects multimorbidity as a major cause for diminished individual resilience. But it may also contribute by triggering side effects, which in turn trigger effects on morbidity [48].

### Advantage-of-treatment markers

Therapy of *benign prostatic hypertrophy* was ongoing in 51 of the 1518 analyzed SHIP participants. These subjects were mainly taking medication, i.e. 5-alpha reductase inhibitors, which hinder the transformation of testosterone into the more potent dihydrotestosterone. The prevalence of benign prostatic hyperplasia is positively related to increasing age [49, 50]. The protective effect of its treatment by medication may be due to reduction of prostate cancer risk [51]. However, our observation may also confirm a recent paradigm shift. Up to a few years ago, hormone replacement therapy by testosterone was considered as an anti-aging treatment, and mainstream thinking may have suppressed dissenting studies. However, testosterone replacement may actually be harmful and low testosterone levels might not be causal for mortality, but might simply reflect a poor health status [52].

In line with previous studies [53], subjects with *treatment for dyslipidemia* had decreased mortality rates in our models. The medications prescribed were mainly statins (N = 64) and fibrates (N = 16). Statins in particular have been studied in large cohorts. Their mechanism of action was attributed to lowering LDL cholesterol [54], while the overall benefit on mortality was shown to occur despite a higher risk of diabetes [55]. Besides lowering hyperlipidemia, both types of medications also have an anti-inflammatory effect [56], which may dampen aging-related inflammation and thus reduce mortality.

For both advantage-of-treatment risk markers, it is furthermore possible that patients seek and find more medical attention, allowing for earlier diagnosis and treatment of other diseases. A well-known example is the survival advantage of older women who had stage 1 breast cancer compared to controls [57]; these survivors of breast cancer may also feature higher resilience in general.

### Blood/urine-based inconclusive-causation markers

High *fibrinogen* levels were already found to predict mortality in previous studies [6, 58, 59], the latter being based on SHIP data. Fibrinogen reflects systemic inflammation, and it is also a general risk marker for arterial and especially coronary and cerebral embolization [60–62]. On the other hand, since aging drives inflammatory processes in older people [21, 63], it is plausible that high fibrinogen levels are also a consequence of advanced age, so that high fibrinogen levels are most likely both cause and consequence of aging processes.

The number of *red blood cells* (RBC) is a marker for oxygen transport capacity. A higher RBC count indicates higher oxygen availability for body physiology and function; a lower RBC count indicates anemia. In fact, a reduction in mortality associated with high RBC counts was found by our group when analyzing cross-sectional data of 30 strains of mice from the Jackson lab [64]. RBC counts are a good example for inconclusive causation, as anemia can be both a consequence of an aging hematopoietic system, and a cause of frailty and subsequent mortality [65].

The *albumin/creatinine ratio* is a chronic kidney disease marker, and it is a well-validated marker of aging and disease processes because several common diseases, e.g. diabetes and hypertension, ultimately trigger kidney malfunction and failure [66–68]. Specifically, a moderately increased *albumin/creatinine ratio* often indicates the beginning of chronic kidney disease, and high levels indicate severe kidney disease [68]. Taking the *albumin/creatinine ratio* as a proxy of chronic kidney dysfunction, we further postulate a mutual relationship between aging processes, diseases and kidney dysfunction, in which it is not straightforward to attribute causality.

We found a protective effect with increased IGF-1 (Insulin-like Growth Factor-1) concentrations. In line with previous analyses of SHIP data [69], IGF-1 values decreased with increasing age. This decrease may be part of a general suppression of endocrine and energy pathways as a consequence of aging [70]. It was also found in SHIP data that low values of an IGF binding protein, IGFBP-3, were associated with increased levels of periodontitis [71]. Moreover, a protective role of IGF-1 was suggested for vascular aging [72]. On the other hand, IGF-1 is considered to be an important contributor to the aging process, and inhibition of IGF and the entire IGF-pathway is known to increase lifespan in several animal models [73], suggesting a causal relationship that is supported by genetic data [74]. However, considering older people but not centenarians, the effect of IGF-1 may change from detrimental to protective as people age. Similar to the case of BMI, we observed a beneficial effect of growth-related processes at older age. This role-change [64] may reflect the obesity paradox [75] in elderly people (but not centenarians), where overweight (but not obesity) enhances survival, probably due to robustness advantages in case of infection and hospitalization. Overall, in terms of phenotype as well as in terms of the underlying pathways, there will be tradeoffs between regenerative capacity and skeletal muscle growth on one hand, and cancer and hyperfunction on the other hand [76].

High serum *calcium* levels were already found to increase mortality in a variety of studies [77], in which participants were almost exclusively recruited at mid-age (i.e. not at very old age). Hypercalcemia often occurs due to malignancy or hyperparathyroidism [78], but can also be caused by excessive skeletal calcium release or decreased renal calcium excretion [79]. High levels of calcium also increase the risk of myocardial infarction [77, 80, 81]. When inspecting the calcium values in detail, we noted that in most age groups, except in the oldest women aged 70–79 years, calcium levels were higher in non-survivors compared to survivors (data not shown). Our group already observed the same role-change as a trend in mouse ([64], Fig 6 therein). More importantly, in the Newcastle 85+ study, for which very old people were recruited, calcium values in the *lowest* percentile were associated with a higher mortality risk [7]. Furthermore, the three studies summarized by Cohen et al [4] are also dominated by very old participants, and accordingly, *low* calcium values were found to be detrimental. Thus, in our data we found that high calcium levels increased mortality, while mortality may still be reduced in very old people, as shown by others. The reason for this ambiguous role of calcium may be due to the complex regulation of calcium levels, resulting in both too high and too low calcium levels being detrimental. This is illustrated by data on vitamin D3 hyper- or hypoactivity [82]: In a model of D3 hyperactivity (induced by dysfunctional klotho, which acts as a vitamin D3 synthesis antagonist), massive vascular calcification ensues, due to calcium reaching its solubility product limit with phosphate—resulting in early death of the animals. By contrast, D3 deficiency is known to be associated not only with osteoporosis, but also vascular nephropathy, increased risk of hypertension, and even cognitive dysfunction [83, 84]. Considering the above-cited studies, the high risk of mortality with high calcium could be interpreted as an increased risk of tissue calcification (including vessels) in the younger population, especially in females, whereas oldest individuals prone to diseases caused by excess calcium are no longer frequently represented, because they already died. The remainder then suffers from vitamin D3 deficiency, lack of calcium, and hence an increased mortality due to this factor (again mainly in the female population).

The *mean corpuscular hemoglobin concentration* (MCHC) is only featured by the AIC model. It is the ratio of hemoglobin and hematocrit, which were also included in the starting set of variables. MCHC is a marker of the individual hemoglobin loading of erythrocytes, and, if low, could be interpreted as an indication of iron deficiency, particularly if the erythrocyte (i.e. RBC) count is also low, which would make it a marker of iron-deficiency anemia (which is often caused by inconspicuous gastrointestinal bleeding), rendering a low MCHC a hazard, in line with the MCHC decrease in older people [4]. Hemoglobin is also included in the AIC model, though statistically insignificant. In fact, while MCHC had no significant correlation with chronological age, both hemoglobin and hematocrit were slightly but significantly correlated in opposite directions for females (0.12 and 0.14, respectively) versus males (-0.28 and -0.28, respectively). This sex-specific difference could be due to the fact that males usually have a higher hematocrit value to start with.

### Contribution of risk markers to mortality and their correlation with chronological age

Usually, predictive markers with a negative influence on survival are expected to drive mortality in stronger ways, the older a person becomes; and vice versa. In our data, all markers with a significantly increased hazard ratio also featured a significantly positive correlation with chronological age, except for smoking status and calcium (see Table 4). In turn, all predictive markers with a significantly protective hazard ratio featured a significantly negative correlation with chronological age, except for the advantage-of-treatment markers (see Table 4). Specifically, treatment of dyslipidemia had a protective impact on mortality even though treatment frequency increased as people aged. On the other hand, ex-smokers still bore an increased hazard. Finally, in case of calcium, we already noted that its role may switch from detrimental to protective in the course of aging, but its detrimental role in all groups except for the oldest women dominated the mortality analysis.

### Strengths and limitations

Our study draws upon the analysis of mortality data of 1518 participants, including 113 non-survivors predominantly aged 60 to 79 years (83 non-survivors). Twenty-six participants died before the age of 60. Our study included very few so-called “oldest-old” (four participants died at an age of 80 and older), which were under focus in other studies [7, 8]. As noted in the discussion of *IGF-1*, this difference in study population may explain differences in the results of mortality analyses. On the other hand, our study also included younger subjects, covering a wide age range (20–79 years at time of recruitment). Thus, our markers are not expected to fully match the markers associated with mortality in a population of, say, age 70 and older. This also holds true for some of the other studies [4], [6]. Then again, we obtained results similar to [10], who evaluated a similar set of variables as we did in a study of men aged 70 and older, also highlighting smoking, anemia and albumin.

We refrained from time-dependent mortality analysis because adding further time points would have implied yet another serious loss of subjects and events due to data missing specifically for these time points. It is likely that some variables changed in time, and this information may have added value to the mortality analysis. Then again, assuming that changes are small to moderate and not systematic, the baseline variable values are expected to contribute the largest amount of information, since they cover the largest available time span during which mortality risks develop and manifest themselves.

The consistency between the two popular modeling approaches we applied, i.e. Cox proportional hazards modeling using the AIC and the BIC criterion, as well as fulfillment of the proportional hazard assumption for all variables, indicate the high quality of the data. Notably, the BIC criterion is more conservative, but the four variables additionally found by using the AIC criterion were plausible as well. Moreover, two of these four variables, i.e. *red blood cell count* and *serum calcium*, completed the picture as they aligned with the “integrated albuminemia” hypothesis of [4]. On the other hand, we admit that the solidity of our results rests a lot on their confirmation by literature searches, not on their direct replication in similar cohorts. In the introduction we described the most similar cohort studies we are aware of, but none of them matches the age distribution in our cohort very well, and, at the same time, offers a strongly overlapping set of variables. Thus, we would like to leave it to future meta-analyses, aggregating our data with other studies, to single out the most generalizing markers and mechanisms behind mortality.

Finally, given that only 1518 of 4308 participant data records were used due to the completeness requirement posed by the Cox regression method, there is a risk of selection bias in the records without missing data: we cannot exclude the possibility that these records belong to participants that are more compliant not only in their participation in the study, but also with respect to the advice they obtain from their physicians, or other advisors involved in maintaining their health. A comparison of various variables (specifically, the ones from the AIC and BIC models) between included and excluded subjects revealed (as expected) differences between both groups, as described in Table D in S1 File. Exclusion of subjects with missing data and consequential bias is essentially unavoidable due to the tradeoff between the number of subjects whose data we can analyze and the choice of variables for which the data are complete. As described in Methods, based on our specific interest in periodontal disease, we selected *mean probing depth* and *mean attachment loss*. In fact, *mean attachment loss* is the variable that features the lowest proportion of values not missing (that is, the lowest completeness) in the full data set, amounting to 80%. We also lost subjects by including variables known to be related to aging/mortality, such as the *albumin creatinine ratio*, *heart rate* and disease/treatment variables such as treated dyslipidemia, for which completeness ranges between 86% and 90%. Here, as for the periodontal disease variables, some lab tests or exams were not done, or results were not valid. As can be expected, such events are not entirely random. For example, more exams tend to be missed by older participants.

### Considerations of causality and correlation

Considerations of causality contribute to our understanding of risk markers and are important for designing interventions. However, contrasting causality and correlation is a difficult issue in any observational study. Following Howick et al. [85] our main criteria for causality were published mechanistic and parallel evidence. In the framework of the Bradford Hill guidelines of evidence for causality, this mechanistic and parallel evidence amounts to biological plausibility, coherence, consistency and analogy [85]; the detailed investigation of biological gradients would require a more equal distribution of mortality events, but unsurprisingly the current data include few deaths before the age of 60. Mechanistic and parallel evidence are contrasted with direct evidence, which unfolds into experiment, strength and temporality [85]. Given that we report results from an observational study without explicit interventions, cause-and-effect relationships are restricted to temporal relationships. For the established markers, causality is taken for granted based on the current literature. For the disease-related risk markers as well as for most physiological or molecular risk markers, the complexity of the processes underlying aging and disease lets us assume “inconclusive causation”. For risk markers related to”integrated albunemia” (red blood cell count, fibrinogen, albumin-creatinine-ratio, calcium), we are in alignment with [4] who explicitly stated that these markers have “a role in the aging process, though causality is not yet clear”. Finally, advantage-of-treatment markers can be assumed to hint at causal processes, but it is unclear whether the positive influence is due to the drugs taken for therapy, or due to the better monitoring of patients that visit their doctor more frequently than the other study participants.

### Conclusions

Summarizing our observations, we found four kinds of markers. The undisputed risk markers, including chronological age, sex and smoking status, were already firmly established in many studies before. A second kind consisted of established disease-related inconclusive-causation markers, including inferior periodontal status, diabetes mellitus, treated gastritis and the number of drugs taken within the last 7 days. Third, we found that specific treatments were protective such that treated subjects featured better survival than non-treated subjects; these were treatment of benign prostatic hypertrophy and treatment of dyslipidemia. Finally, we found a set of physiological / molecular markers, featuring “inconclusive causation”. Some of these aligned with the”integrated albunemia” model of aging proposed recently [4], which implicated anemia, inflammation and low levels of albumin and calcium as markers of mortality and frailty. In our analysis, anemia, inflammation and albumin were represented by red blood cell counts, fibrinogen, and the albumin/creatinine ratio, respectively. The latter hints at a potential connection between serum and urinary albumin levels [86]. In our study, serum calcium levels predicted mortality as well, but their role was different compared to Cohen et al [4], possibly because our study population was not specifically sampled from very old people.

## Supporting information

S1 File Tables A to D, Figure A. (DOCX)
