# Supplementary material for: Mortality is associated with inflammation, anemia, specific diseases and treatments, and molecular markers
Source: PLoS One. 2017 Apr 19;12(4):e0175909. doi: 10.1371/journal.pone.0175909 (PMC5397036; doi:10.1371/journal.pone.0175909)
Supplement: S1 File — (DOCX) [file pone.0175909.s001.docx]

**Table A. Variables considered for modelling (77 independent variables).**

| **Variable** | **Kind of variable** | **Unit** | **Definition and/or Coding** |
| --- | --- | --- | --- |
| General information |  |  |  |
| Age | quantitative | year | Completed years a survey |
| Sex | categorical |  | “0” if male, “1” if female |
| Socio-demographic variables |  |  |  |
| Marital status | categorical |  | “1” if single and living alone, “2” if married and living together with spouse or if (single / married and living separated from spouse / divorced / widowed) and living together with a partner, “3” if (married and living separated from spouse / divorced) and not living together with a partner, “4” if widowed and not living together with a partner |
| Living in a partnership | categorical |  | “1/Yes” if living together with spouse or partner else “0/No” |
| School education | categorical |  | “1” if less than 10 years, “2” if 10 years and “3” if more than 10 years |
| Household equivalent income | quantitative | € | The net income was obtained by allocating the mean of the respective categories as monetary value (back then Deutschmark). To obtain the monetary value in Euro, the resulting net income was divided by 1.95583. Finally, the household equivalent income represents the net income in Euro divided by the square root of the number of people living in the household. |
| Health-related behavior |  |  |  |
| Smoking status | categorical |  | Never smoker, ex-smoker and current smoker |
| Pack years | quantitative | integer | number of cigarettes smoked per day /20 * number of years the person has smoked |
| Alcohol consumption | quantitative | g/day | Based on the self-reported consumption during the last week |
| Physical activity | categorical |  | “1/Yes” if ≥ 1h of physical training per week during summer or winter else “0/No” |
| Food frequency score | quantitative |  | According to [[1](#_ENREF_1), [2](#_ENREF_2)] |
| Somatometric measurements |  |  |  |
| Body height | quantitative | cm |  |
| Body mass index (BMI) | quantitative | kg/m^2^ | Body weight [kg] / (body height [m])^2 |
| Waist to hip ratio (WHR) | quantitative |  | Waist circumference [cm] / hip circumference [cm] |
| Diseases and overall health |  |  |  |
| Depression | categorical |  | “1/Yes” if persistent sadness and lethargy for ≥ 2 weeks else “0/No” |
| Mental Health Composite Scale Score | quantitative |  | SF-12 sum-score of mental health |
| Physical Health Composite Scale Score | quantitative |  | SF-12 sum-score of physical health |
| Diagnosed asthma | categorical |  | “1/Yes” if present else “0/No” |
| Diabetes mellitus | categorical |  | “1/Yes” if self-reported physician’s diagnosis or treatment with insulin or antidiabetic agents (ATC Code A10*) or non-fasting glucose levels ≥11.1 mmol/l or HbA1c ≥ 6.5% else “0/No” |
| Systolic blood pressure | quantitative | mmHg | Mean of 2nd and 3rd measurement |
| High blood pressure | categorical |  | “1/Yes” if systolic blood pressure ≥140 or diastolic blood pressure ≥90 or antihypertensive medication else “0/No” |
| Metabolic syndrome | categorical |  | “1/Yes” if present else “0/No” |
| Treated dyslipidemia | categorical |  | “1/Yes” if present else “0/No” |
| Treated kidney disease | categorical |  | “1/Yes” if present else “0/No” |
| Treated arthritis | categorical |  | “1/Yes” if present else “0/No” |
| Treated bronchitis | categorical |  | “1/Yes” if present else “0/No” |
| Treated gastritis | categorical |  | “1/Yes” if present else “0/No” |
| Treated osteoporosis | categorical |  | “1/Yes” if present else “0/No” |
| Treated thrombosis | categorical |  | “1/Yes” if present else “0/No” |
| Treated thyroid disease | categorical |  | “1/Yes” if present else “0/No” |
| Heart rate | quantitative | bpm |  |
| Stationary treated heart attack | categorical |  | “1/Yes” if applicable else “0/No” |
| Stationary treated stroke | categorical |  | “1/Yes” if applicable else “0/No” |
| Oral health |  |  |  |
| Number of teeth | quantitative | integer | Excluding third molars (max. 28) |
| Mean attachment loss | quantitative | mm | Mean over all measured distances from the cemento-enamel junction to the bottom of the periodontal pocket |
| Mean pocket probing depth | quantitative | mm | Mean over all measured distances from the gingival margin to the base of the periodontal pocket |
| Medication |  |  |  |
| Number of drugs taken in last 7 days | quantitative | integer |  |
| Antiandrogens | categorical |  | “1/Yes” if prescribed medication includes ATC Code (G03H*) else “0/No” |
| Anticoagulants | categorical |  | “1/Yes” if prescribed medication includes ATC Code (B01*, B03AA*) else “0/No” |
| Antiemetics | categorical |  | “1/Yes” if prescribed medication includes ATC Code (A03F*) else “0/No” |
| Antigout preparations | categorical |  | “1/Yes” if prescribed medication includes ATC Code (M04A*) else “0/No” |
| Antihypertensives | categorical |  | “1/Yes” if prescribed medication includes ATC Code (C02A*, C03C*, C03E*, C09BA*, C07A*, C08C*, C09AA*, C09CA*) else “0/No” |
| Anti-inflammatory and antirheumatic products, non-steroids | categorical |  | “1/Yes” if prescribed medication includes ATC Code (M01A*) else “0/No” |
| Calcium supplements | categorical |  | “1/Yes” if prescribed medication includes ATC Code (A12A*) else “0/No” |
| Drugs for acid related disorders | categorical |  | “1/Yes” if prescribed medication includes ATC Code (A02*) else “0/No” |
| Drugs used in benign prostatic hypertrophy | categorical |  | “1/Yes” if prescribed medication includes ATC Code (G04C*) else “0/No” |
| Estrogens | categorical |  | “1/Yes” if prescribed medication includes ATC Code (G03C*) else “0/No” |
| Estrogens and progestogens in combination | categorical |  | “1/Yes” if prescribed medication includes ATC Code (G03F*) else “0/No” |
| HMG-CoA reductase inhibitors | categorical |  | “1/Yes” if prescribed medication includes ATC Code (C10AA*) else “0/No” |
| Medication for the respiratory system | categorical |  | “1/Yes” if prescribed medication includes ATC Code (R03*, R05CB*) else “0/No” |
| Sex hormones and modulators of the genital system | categorical |  | “1/Yes” if prescribed medication includes ATC Code (G03*) else “0/No” |
| Laboratory variables |  |  |  |
| HbA1c | quantitative | % |  |
| Triglycerides | quantitative | mmol/l |  |
| High density lipoprotein (HDL) - Cholesterol | quantitative | mmol/l |  |
| Low Density Lipoprotein (LDL) - Cholesterol | quantitative | mmol/l |  |
| Cholesterol - high density lipoprotein ratio | quantitative |  | Total cholesterol [mmol/l] / high density lipoprotein – cholesterol [mmol/l] |
| Lipase | quantitative | µmol/sl |  |
| Lipoprotein A | quantitative | mg/l |  |
| Apolipoprotein A1 | quantitative | g/l |  |
| Apolipoprotein B | quantitative | g/l |  |
| Ferritin | quantitative | µg/l |  |
| Serum calcium | quantitative | mmol/l |  |
| Serum magnesium concentration | quantitative | mmol/l |  |
| Serum potassium concentration | quantitative | mmol/l |  |
| Serum sodium concentration | quantitative | mmol/l |  |
| Free T4 | quantitative | pmol/l |  |
| IGF-1 (insulin-like growth factor – 1) | quantitative | ng/ml | Nichols Advantage |
| Albumin/creatinine ratio, mg/mmol | quantitative | mg/g | Albumin [mg/l] / (Creatinine [mmol/l] *0.113118 |
| Glomerular filtration rate | quantitative | ml/min per 1.73m^2^ | Men: 186*(( serum creatinine*0.0113118)^-1,154)*(age^-0.203)  Women: 186*(( serum creatinine*0.0113118)^-1,154)*(age^-0.203)*0.742 |
| Cyclic citrullinated peptide (CCP) | quantitative | U/ml | Immunocap 250 |
| C-Reactive Protein (CRP) | quantitative | mg/l |  |
| Fibrinogen | quantitative | g/l | Clauss method |
| White blood cell count | quantitative | Gpt/l |  |
| Red blood cell count | quantitative | Tpt/l |  |
| Platelet count (PLT) | quantitative | Gpt/l |  |
| Hemoglobin | quantitative | mmol/l |  |
| Mean corpuscular hemoglobin concentration (MCHC) | quantitative | mmol/l |  |

**Table B. Comparison of the AIC and the BIC model according to their hazard ratios and p-values. Z-standardized continuous variables are marked by (^a^).**

| **Variable** | **AIC model** | | **BIC model** | |
| --- | --- | --- | --- | --- |
|  | **HR (95% CI)** | **P-value** | **Hazard Ratio** | **P-value** |
| Age, years | 1.09 (1.06; 1.12) | <0.01 | 1.09 (1.06; 1.11) | <0.01 |
| Female sex (Ref. male sex) | 0.47 (0.28; 0.79) | <0.01 | 0.51 (0.32; 0.80) | <0.01 |
| Smoking status (Ref. Never smoker) |  |  |  |  |
| Ex-Smoker | 1.51 (0.87; 2.62) | 0.14 | 1.44 (0.85; 2.43) | 0.17 |
| Smoker | 3.55 (1.89; 6.65) | <0.01 | 3.37 (2.13; 6.55) | <0.01 |
| Household equivalent income, € ^a^ | 0.77 (0.59; 1.01) | 0.06 | - | - |
| SF-12 sum-score of physical health ^a^ | 0.87 (0.73; 1.03) | 0.11 | - | - |
| Mean attachment loss, mm ^a^ | 1.47 (1.23; 1.75) | <0.01 | 1.46 (1.23; 1.72) | <0.01 |
| Depression, yes | 1.65 (0.86; 3.15) | 0.13 | - | - |
| Diabetes mellitus, yes | 1.63 (1.02; 2.60) | 0.04 | - | - |
| Treated dyslipidemia, yes | 0.29 (0.16; 0.55) | <0.01 | 0.40 (0.22; 0.70) | <0.01 |
| Treated gastritis, yes | 2.48 (1.31; 4.71) | 0.01 | 2.48 (1.34; 4.61) | <0.01 |
| Treated osteoporosis, yes | 0.48 (0.18; 1.28) | 0.14 | - | - |
| Number of drugs taken within the last 7 days | 1.17 (1.05; 1.29) | <0.01 | 1.20 (1.11; 1.30) | <0.01 |
| Antihypertensives, yes | 0.69 (0.42; 1.12) | 0.13 | - | - |
| Sex hormones and modulators of the genital system, yes | 0.38 (0.09; 1.65) | 0.19 | - | - |
| HMG CoA reductase inhibitors, yes | 1.70 (0.89; 3.26) | 0.11 | - | - |
| Drugs used in benign prostatic hypertrophy, yes | 0.40 (0.21; 0.77) | 0.01 | 0.38 (0.20; 0.73) | 0.01 |
| Serum calcium, mmol/l ^a^ | 1.43 (1.15; 1.77) | <0.01 | - | - |
| Ferritin, µg/l ^a^ | 0.85 (0.69; 1.04) | 0.12 | - | - |
| IGF-1, ng/ml ^a^ | 0.63 (0.46; 0.85) | <0.01 | 0.69 (0.52; 0.92) | 0.01 |
| Fibrinogen, g/l ^a^ | 1.53 (1.30; 1.78) | <0.01 | 1.46 (1.27; 1.68) | <0.01 |
| Albumin/creatinine ratio, mg/mmol ^a^ | 1.16 (1.05; 1.27) | <0.01 | 1.16 (1.06; 1.26) | <0.01 |
| Red blood cell count, Tpt/l ^a^ | 0.54 (0.37; 0.78) | <0.01 | - | - |
| Hemoglobin, mmol/l ^a^ | 1.44 (0.97; 2.15) | 0.07 | - | - |
| Mean corpuscular hemoglobin concentration, mmol/l * | 0.67 (0.54; 0.84) | <0.01 | - | - |

HR, Hazard ratio; CI, confidence interval

**Table C. Comparison of the AIC and the BIC model (without z-standardization) by their Hazard ratios and p-values.**

| **Variable** | **AIC model** | | **BIC model** | |
| --- | --- | --- | --- | --- |
|  | **HR (95% CI)** | **P-value** | **HR (95% CI)** | **P-value** |
| Age, years | 1.09 (1.06; 1.12) | <0.01 | 1.09 (1.06; 1.11) | <0.01 |
| Female sex (Ref. male sex) | 0.47 (0.28; 0.79) | <0.01 | 0.51 (0.32; 0.80) | <0.01 |
| Smoking status (Ref. Never smoker) |  |  |  |  |
| Ex-Smoker | 1.51 (0.87; 2.62) | 0.14 | 1.44 (0.85; 2.43) | 0.17 |
| Smoker | 3.55 (1.89; 6.65) | <0.01 | 3.37 (2.13; 6.55) | <0.01 |
| Household equivalent income, € | 0.999 (0.999; 1.000) | 0.06 | - | - |
| SF-12 sum-score of physical health | 0.98 (0.96; 1.00) | 0.11 | - | - |
| Mean attachment loss, mm | 1.22 (1.11; 1.33) | <0.01 | 1.21 (1.11; 1.32) | <0.01 |
| Depression, yes | 1.65 (0.86; 3.15) | 0.13 | - | - |
| Diabetes mellitus, yes | 1.62 (1.02; 2.60) | 0.04 | - | - |
| Treated dyslipidemia, yes | 0.29 (0.16; 0.55) | <0.01 | 0.40 (0.22; 0.70) | <0.01 |
| Treated gastritis, yes | 2.48 (1.31; 4.71) | 0.01 | 2.48 (1.34; 4.61) | <0.01 |
| Treated osteoporosis, yes | 0.48 (0.18; 1.28) | 0.14 | - | - |
| Number of drugs taken within the last 7 days | 1.16 (1.05; 1.29) | <0.01 | 1.20 (1.11; 1.30) | <0.01 |
| Antihypertensives, yes | 0.69 (0.42; 1.12) | 0.13 | - | - |
| Sex hormones and modulators of the genital system, yes | 0.38 (0.09; 1.65) | 0.19 | - | - |
| HMG CoA reductase inhibitors, yes | 1.70 (0.89; 3.26) | 0.11 | - | - |
| Drugs used in benign prostatic hypertrophy, yes | 0.40 (0.21; 0.77) | 0.01 | 0.38 (0.20; 0.73) | 0.01 |
| Serum calcium, mmol/l | 25.65 (3.67; 179.44) | <0.01 | - | - |
| Ferritin, µg/l | 0.999 (0.997; 1.0004) | 0.12 | - | - |
| IGF-1, ng/ml | 0.992 (0.987; 0.997) | <0.01 | 0.994 (0.989; 0.999) | 0.01 |
| Fibrinogen, g/l | 1.87 (1.49; 2.36) | <0.01 | 1.75 (1.42; 2.16) | <0.01 |
| Albumin/creatinine ratio, mg/mmol | 1.002 (1.001; 1.003) | <0.01 | 1.002 (1.001; 1.003) | <0.01 |
| Red blood cell count, Tpt/l | 0.22 (0.09; 0.54) | <0.01 | - | - |
| Hemoglobin, mmol/l | 1.59 (0.96; 2.63) | 0.07 | - | - |
| Mean corpuscular hemoglobin concentration, mmol/l | 0.51 (0.35; 0.74) | <0.01 | - | - |

HR, Hazard ratio; CI, confidence interval; Hazard ratios of continuous variables refer to a 1-unit increase of the independent variable

**Table D. Baseline characteristics of included and excluded subjects.**

|  | Included subjects  (N=1518) | Excluded subjects  (N_max_=2790) | P value * |
| --- | --- | --- | --- |
| Variables selected into final AIC or BIC models |  |  |  |
| Age, years | 46.3±15.5 | 51.7±16.5 (N=2790) | <0.001 |
| Female sex | 51.5% | 50.5% (N=2790) | 0.56 |
| Household equivalent income, € | 981.4±483.3 | 958.0±491.1 (N=2539) | 0.06 |
| Smoking status |  |  |  |
| Never smoker | 35.6% | 36.4% |  |
| Ex-smoker | 32.7% | 34.3% |  |
| Smoker | 31.8% | 29.3% (N=2790) | 0.10 |
| SF-12 sum-score of physical health | 49.1±8.2 | 47.4±9.4 (N=2471) | <0.001 |
| Mean attachment loss, mm | 2.65 | 2.61 (N=2049) | 0.81 |
| Depression, yes | 12.5% | 13.1% (N=2732) | 0.57 |
| Diabetes mellitus, yes | 8.2% | 12.5% (N=2775) | <0.001 |
| Treated dyslipidemia, yes | 10.9% | 16.0% (N=2367) | <0.001 |
| Treated gastritis, yes | 5.4% | 5.6% (N=2745) | 0.87 |
| Treated osteoporosis, yes | 3.1% | 4.2% (N=2652) | 0.09 |
| Number of drugs taken within the last 7 days | 1.77±2.13 | 2.33±2.66 (N=2773) | <0.001 |
| Antihypertensives, yes | 21.9% | 29.9% (N=2773) | <0.001 |
| Sex hormones and modulators of the genital system, yes | 17.8% | 13.0% (N=2790) | <0.001 |
| HMG CoA reductase inhibitors, yes | 5.6% | 7.0% (N=2773) | 0.08 |
| Drugs used in benign prostatic hypertrophy, yes | 3.4% | 3.1% (N=2790) | 0.686 |
| Serum calcium, mmol/l | 2.4±0.1 | 2.4±0.1 (N=2772) | <0.001 |
| Ferritin, μg/l | 96.6±105.6 | 111.2±144.6 (N=2770) | <0.001 |
| IGF-1, ng/ml | 150.2±59.4 | 136.8±56.1 (N=2561) | <0.001 |
| Fibrinogen, g/l | 2.9±0.7 | 3.0±72(N=2766) | <0.001 |
| Urine albumin/creatinine ratio, mg/mmol | 21.9±68.0 | 30.8±86.86 (N=2276) | <0.001 |
| Red blood cell count, Tpt/l | 4.43±0.4 | 4.42±0.43 (N=2773) | <0.001 |
| Hemoglobin, mmol/l | 8.4±0.8 | 8.5±0.8 (N=2771) | 0.13 |
| Mean corpuscular hemoglobin concentration, mmol/l | 21.2±0.6 | 21.2±0.6 (N=2772) | <0.001 |
| Additional variables |  |  |  |
| School education |  |  |  |
| <10 years | 31.7% | 44.7% |  |
| 10 years | 49.7% | 40.3% |  |
| >10 years | 18.6% | 15.1% (N=2760) | <0.001 |
| Body mass index, kg/m^2^ | 26.9±4.8 | 27.5±4.7 (N=2780) | 0.003 |
| HbA1c, % | 5.3±0.9 | 5.5±1.0 (N=2767) | <0.001 |
| Hypertension, yes | 48.0% | 54.9% (N=2775) | <0.001 |
| hsCRP, mg/l | 2.8±6.1 | 3.1±5.1 (N=2466) | 0.004 |
| Number of teeth | 20.7±7.0 | 15.8±10.4 (N=2770) | <0.001 |

Data are presented as mean ± standard deviation or percentages. The number of excluded subjects with non-missing data is additionally provided in brackets. * Differences in variable distributions between included and excluded subjects were tested by Mann-Whitney-U-test or Chi- squared test. IGF-1, Insulin like growth factor 1; hsCRP, high -sensitive C-reactive protein; HbA1c, hemoglobin A1c.


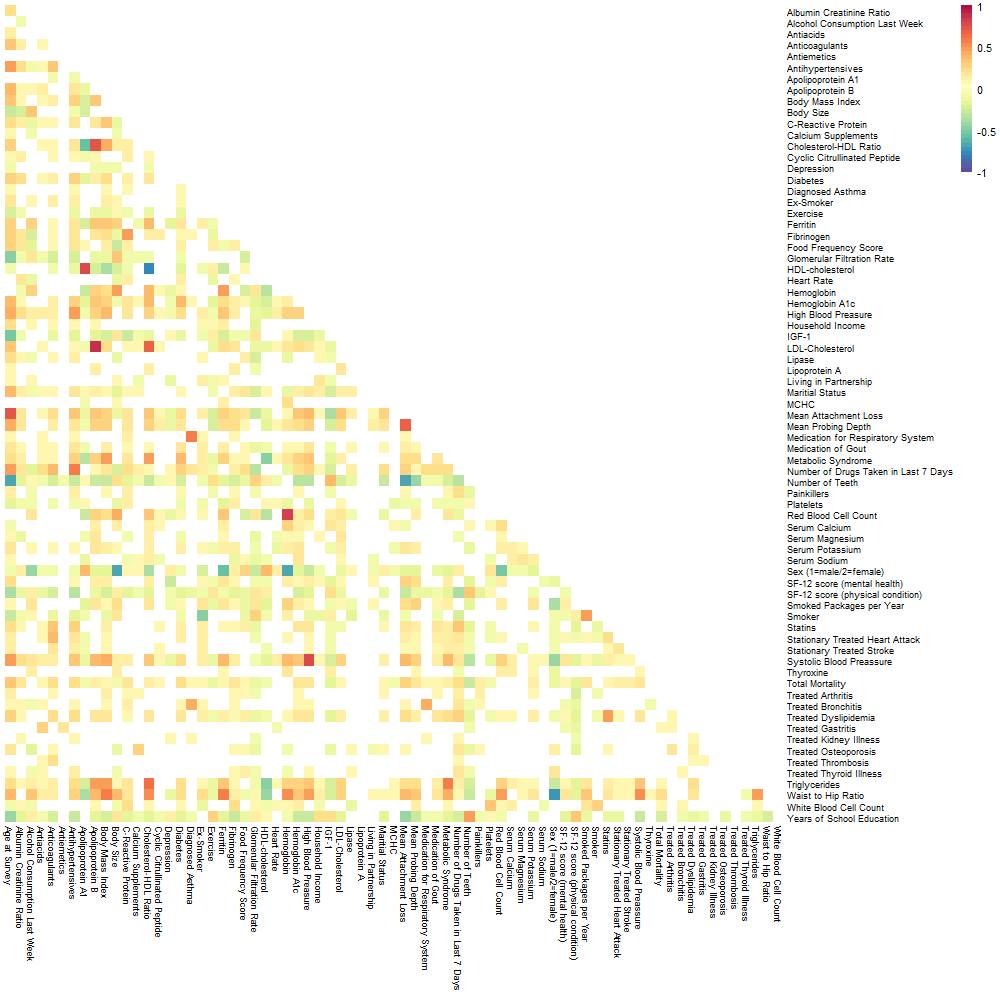
 **Figure A. Spearman’s correlation matrix for variables considered for the model selection process.** Red colors (with intensity depending on correlation value) indicate positive correlations (r_Sp_>0), while green to blue colors indicate negative correlations (r_Sp_<0). Missing correlations are colored in white.

**References**

1. Winkler G, Doring A, Keil U. [Mealtime patterns in a southern German population. Results from the WHO MONICA 1984/1985 Augsburg nutritional survey project]. Z Ernahrungswiss. 1995;34(1):2-9. Epub 1995/03/01. PubMed PMID: 7785293.

2. Luedemann J, Schminke U, Berger K, Piek M, Willich SN, Doring A, et al. Association between behavior-dependent cardiovascular risk factors and asymptomatic carotid atherosclerosis in a general population. Stroke. 2002;33(12):2929-35. Epub 2002/12/07. PubMed PMID: 12468793.
